# Resilient Capacity-Aware Routing

**DOI:** 10.1007/978-3-030-72016-2_22

**Published:** 2021-03-01

**Authors:** Stefan Schmid, Nicolas Schnepf, Jiří Srba

**Affiliations:** 8 Eindhoven University of Technology, Eindhoven, The Netherlands; 9 Aalborg University, Aalborg East, Denmark; 10 Faculty of Computer Science, University of Vienna, Vienna, Austria; 11 Department of Computer Science, Aalborg University, Aalborg, Denmark

## Abstract

To ensure a high availability, communication networks provide resilient routing mechanisms that quickly change routes upon failures. However, a fundamental algorithmic question underlying such mechanisms is hardly understood: how to verify whether a given network reroutes flows along *feasible* paths, without violating capacity constraints, for up to *k* link failures? We chart the algorithmic complexity landscape of resilient routing under link failures, considering shortest path routing based on link weights as e.g. deployed in the ECMP protocol. We study two models: a *pessimistic* model where flows interfere in a worst-case manner along equal-cost shortest paths, and an *optimistic* model where flows are routed in a best-case manner, and we present a complete picture of the algorithmic complexities. We further propose a strategic search algorithm that checks only the critical failure scenarios while still providing correctness guarantees. Our experimental evaluation on a benchmark of Internet and datacenter topologies confirms an improved performance of our strategic search by several orders of magnitude.

## 1 Introduction

Routing and traffic engineering are most fundamental tasks in a communication network. Internet Service Providers (ISPs) today use several sophisticated strategies to efficiently provision their backbone network to serve intra-domain traffic. This is challenging as in addition to simply providing reachability, routing protocols should also account for capacity constraints: to meet quality-of-service guarantees, congestion must be avoided. Intra-domain routing protocols are usually based on shortest paths, and in particular the Equal-Cost-MultiPath (ECMP) protocol [24]. Flows are split at nodes where several outgoing links are on shortest paths to the destination, based on per-flow static hashing [7, 30]. In addition to default routing, most modern communication networks also provide support for *resilient routing*: upon the detection of a link failure, the network nodes quickly and collaboratively recompute the new shortest paths [21].

However, today, we still do not have a good understanding of the algorithmic complexity of shortest path routing subject to capacity constraints, especially under failures. In particular, in this paper we are interested in the basic question: "Given a capacitated network based on shortest path routing (defined by link weights), can the network tolerate up to $k$ link failures without violating capacity constraints?" Surprisingly only little is known about the complexity aspects.

* srba@cs.aau.dk

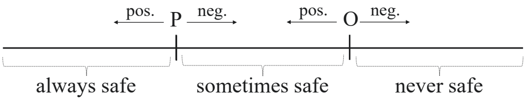

Fig. 1: Classification of possible network situations

| | Pessimistic | Optimistic |
|---|---|---|
| Splittable | NL-complete | P-complete |
| Nonsplit. | NL-complete | NP-complete |

(a) Without link failures ($k = 0$)

| | Pessimistic | Optimistic |
|---|---|---|
| Splittable | co-NP-complete | co-NP-complete |
| Nonsplit. | co-NP-complete | $\Pi_2^P$-complete |

(b) With link failures ($k \geq 0$)

Fig. 2: Summary of complexity results for capacity problems

*Our Contributions.* We provide a complete characterization of the algorithmic complexity landscape of resilient routing and introduce two basic models of how traffic is distributed across the multiple shortest paths. A **pessimistic (P)** one where flows add up in a worst-case manner; if a network is resilient in the pessimistic model, it is guaranteed that routing succeeds along *any* shortest path without overloading links. In the **optimistic (O)** model flows add up in a best-case manner; if a network is resilient in the optimistic model, it *may be* that the specific routing does not overload the links. The two models hence cover the two extremes in the spectrum and alternative routing schemes, e.g., (pseudo)random routing hence lies in between. Figure 1 illustrates the situations that can arise in a network: depending on the scenario, pessimistic (P) or optimistic (O), and whether the routing feasibility test is positive or negative, we can distinguish between three regimes. (1) If routing is feasible even in the pessimistic case, then flows can be safely forwarded by any routing policy without violating any capacity constraints. (2) If the pessimistic test is negative but positive in the optimistic case, then further considerations are required to ensure that flows use the feasible paths (e.g., a clever routing algorithm to find the suitable paths is needed). (3) If even the optimistic test is negative then no feasible routing solution exists; to be able to successfully route flows in this case, we need to change the network characteristics, e.g., to increase the link capacities.

We further distinguish between **splittable (S)** and **nonsplittable (N)** flows, and refer to the four possible problems by **PS**, **PN**, **ON**, and **OS**. Our main complexity results are summarized in Figure 2. We can see that without link failures (Figure 2a), the problems are solvable in polynomial time, except for the ON problem that becomes NP-complete. Moreover, the pessimistic variants of the problem can be solved even in nondeterministic logarithmic space, implying that they allow for efficient parallelization [33]. On the other hand, the optimistic splittable problem is hard for the class P. For the problems with link failures (Figure 2b) the complexity increases and the problems become co-NP-complete, apart from the ON problem that becomes more difficult to solve and is complete for the second level of the polynomial hierarchy [33].

The high computational complexity of the instances with link failures may indicate that a brute-force search algorithm exploring all failure scenarios is needed to verify whether routing is feasible. However, we present a more efficient solution, by defining a partial ordering on the possible failure scenarios with the property that for the pessimistic model, we only need to explore the minimum failure scenarios, and for the optimistic model, it is sufficient to explore the maximum failure scenarios. We present an efficient *strategic search* algorithm implementing these ideas, formally prove its correctness, and demonstrate the practical applicability of strategic search on a benchmark of Internet and datacenter topologies. In particular, we find that our algorithm achieves up to several orders of magnitude runtime savings compared to the brute-force search.

*Related Work.* Efficient traffic routing has received much attention in the literature, and there also exist empirical studies on the efficiency of ECMP deployments, e.g., in Internet Service Provider Networks [17] or in datacenters [22]. A systematic algorithmic study of routing with ECMP is conducted by Chiesa et al. in [10]. The authors show that in the splittable-flow model [16], even approximating the optimal link-weight configuration for ECMP within any constant factor is computationally intractable. Before their work, it was only known that minimizing congestion is NP-hard (even to just provide "satisfactory" quality [2] and also under path cardinality constraints [5]) and cannot be approximated within a factor of 3/2 [19]. For specific topologies the authors further show that traffic engineering with ECMP remains suboptimal and computationally hard for hypercube networks. We significantly extend these insights into the algorithmic complexity of traffic engineering and introduce the concept of pessimistic and optimistic variants of routing feasibility and provide a complete characterization of the complexity of routing subject to *capacity constraints*, also in scenarios with *failures*. Accounting for failures is an important aspect in practice [13, 31] but has not been studied rigorously in the literature before; to the best of our knowledge, so far there only exist heuristic solutions [18] with some notable exceptions such as Lancet [8] (which however does not account for congestion). We propose to distinguish between optimistic and pessimistic flow splitting; existing literature typically revolves around the optimistic scenario.

We note that while we focus on IP networks (and in particular shortest path routing and ECMP), there exist many interesting results on the verification and reachability testing in other types of networks and protocols, including BGP [4, 15], MPLS [25, 38], OpenFlow [1] networks, or stateful networks [29, 32, 41]. While most existing literature focuses on verifying logical properties, such as reachability without considering capacity constraints, there also exist first works dealing with quantitative properties [20, 26, 29].

## 2 Network with Capacities and Demands

We shall now define the model of network with link capacities and flow demands and formally specify the four variants of the resilient routing problem. Let $\mathbb{N}$ be the set of natural numbers and $\mathbb{N}^0$ the set of nonnegative integers.

**Definition 1 (Network with Capacities and Demands).** *A* Network with Capacities and Demands *(NCD) is a triple $N = (V, C, D)$ where $V$ is a finite set of* nodes*, $C : V \times V \mapsto \mathbb{N}^0$ is the* capacity function *for each network edge (capacity 0 implies the absence of a network link), and $D : V \times V \mapsto \mathbb{N}^0$ is the end-to-end* flow demand *between every pair of nodes such that $D(v, v) = 0$ for all $v \in V$ (demand 0 means that there is no flow).*

Let $N = (V, C, D)$ be an NCD. A *path* from $v_1$ to $v_n$ where $v_1, v_n \in V$ is any nonempty sequence of nodes $v_1 v_2 \cdots v_n \in V^+$ such that $C(v_i, v_{i+1}) > 0$ for all $i$, $1 \leq i < n$. Let $s, t \in V$. By $Paths(s, t)$ we denote the set of all paths from $s$ to $t$. Let $\pi \in Paths(s, t)$ be a path in $N$ such that $\pi = v_1 v_2 \ldots v_n$. An *edge* is a pair of nodes $(v, v') \in V \times V$ such that $C(v, v') > 0$. We write $(v, v') \in \pi$ whenever $(v, v') = (v_i, v_{i+1})$ for some $i$, $1 \leq i < n$.

Routes in an NCD are traditionally determined by annotating the links with weights and employing shortest path routing (e.g. ECMP). In case of multiple shortest paths, traffic engineers select either one of the shortest paths or decide to split the flow among the different shortest paths for load-balancing purposes. When one or multiple links fail, the set of shortest paths may change and the routes need to be updated. The weight assignment is usually provided by the network operators and is primarily used for traffic engineering purposes.

**Definition 2 (Weight Assignment).** *Let $N = (V, C, D)$ be an NCD. A* weight assignment *on $N$ is a function $W : V \times V \mapsto \mathbb{N} \cup \{\infty\}$ that assigns each link a positive weight where $C(v, v') = 0$ implies that $W(v, v') = \infty$ for all $v, v' \in V$.*

Assume now a fixed weight assignment for a given NCD $N = (V, C, D)$. Let $\pi = v_1 v_2 \cdots v_n \in V^+$ be a *path* from $v_1$ to $v_n$. The *weight* of the path $\pi$ is denoted by $W(\pi)$ and defined by $W(\pi) = \sum_{i=1}^{n-1} W(v_i, v_{i+1})$. Let $s, t \in V$. The set of shortest paths from $s$ to $t$ is defined by $SPaths(s, t) = \{\pi \in Paths(s, t) \mid W(\pi) \neq \infty$ and $W(\pi) \leq W(\pi')$ for all $\pi' \in Paths(s, t)\}$. As the weights are positive, all shortest paths in the set $SPaths(s, t)$ are acyclic and hence the set is finite (though of possibly exponential size).

For a given NCD $N$ and a set of failed links $F$, we can now define the NCD $N^F$ where all links from $F$ are removed.

**Definition 3.** *Let $N = (V, C, D)$ be an NCD with weight assignment $W$, and let $F \subseteq V \times V$ be a set of failed links. We define the* pruned *NCD $N^F = (V, C^F, D)$ with an updated weight assignment $W^F$ by*

- *$C^F(v, v') = C(v, v')$ and $W^F(v, v') = W(v, v')$ if $(v, v') \notin F$, and*
- *$C^F(v, v') = 0$ and $W^F(v, v') = \infty$ if $(v, v') \in F$.*

By $Paths^F(s, t)$ and $SPaths^F(s, t)$ we denote the sets of the paths and shortest paths between $s$ and $t$ in the network $N^F = (V, C^F, D)$ with $W^F$.

We shall now define a flow assignment that for each nonempty flow demand between $s$ and $t$ and every failure scenario, determines the amount of traffic that should be routed through the shortest paths between $s$ and $t$.

**Definition 4 (Flow Assignment).** *A* flow assignment $f$ *in a capacity network* $N = (V, C, D)$ *with weight assignment* $W$ *and with the set* $F \subseteq V \times V$ *of failed links is a family of functions* $f^F_{s,t} : SPaths^F(s,t) \mapsto [0,1]$ *for all* $s, t \in V$ *where* $D(s,t) > 0$ *such that* $\sum_{\pi \in SPaths^F(s,t)} f^F_{s,t}(\pi) = 1$*. A flow assignment* $f$ *is* nonsplittable *if* $f^F_{s,t}(\pi) \in \{0,1\}$ *for all* $s,t \in V$ *and all* $\pi \in SPaths^F(s,t)$*. Otherwise the flow assignment is* splittable*.*

The notation $[0,1]$ denotes the interval of all rational numbers between 0 and 1 and it determines how the load demand between the nodes $s$ and $t$ is split among the routing paths between the two nodes. A nonsplittable flow assignment assigns the value 1 to exactly one routing path between any two nodes $s$ and $t$. If for a given failure scenario $F$ there is no path between $s$ and $t$ for two nodes with $D(s,t) > 0$, then there is no flow assignment as the network is *disconnected.*

**Definition 5.** *An NCD* $N = (V, C, D)$ *is* connected *for the set of failed links* $F \subseteq V \times V$ *if* $SPaths^F(s,t) \neq \emptyset$ *for every* $s, t \in V$ *where* $D(s,t) > 0$*.*

For a connected NCD, we now define a feasible flow assignment that avoids congestion: the sum of portions of flow demands (determined by the flow assignment) that are routed through each link, may not exceed the link capacity.

**Definition 6 (Feasible Flow Assignment).** *Let* $N = (V, C, D)$ *be an NCD with weight assignment* $W$*. Let* $F \subseteq V \times V$ *be the set of failed links s.t. the network remains connected. A flow assignment* $f$ *is* feasible *if every link* $(v, v') \in V \times V$ *with* $C(v,v') > 0$ *satisfies* $\sum_{\substack{s,t \in V \\ \pi \in SPaths^F(s,t) \\ (v,v') \in \pi}} f^F_{s,t}(\pi) \cdot D(s,t) \leq C(v,v')$*.*

We consider four different variants of the capacity problem.

**Definition 7 (Pessimistic Splittable/Nonsplittable (PS/PN)).** *Given an NCD* $N$ *with a weight assignment and nonnegative integer* $k$*, is it the case that for* every *set* $F$ *of failed links of cardinality at most* $k$*, the network remains connected and* every *splittable/nonsplittable flow assignment on* $N$ *with the set* $F$ *of failed links is feasible?*

**Definition 8 (Optimistic Splittable/Nonsplittable (OS/ON)).** *Given an NCD* $N$ *with a weight assignment and a nonnegative integer* $k$*,* is there *a feasible splittable/nonsplittable flow assignment on* $N$ *for* every *set of failed links* $F$ *of cardinality at most* $k$*?*

A positive answer to the PN capacity problem implies positive answers to both PS and ON problems. A positive answer to either the PS or ON problem implies a positive answer to the OS problem. This is summarized in Figure 3 and it is easy to argue that the hierarchy is strict.

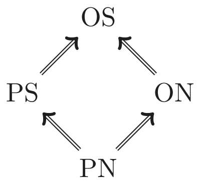

Fig. 3: Hierarchy

## 3 Analysis of Algorithmic Complexity

We now provide the arguments for the upper and lower bounds from Figure 2.

**Algorithm 1** Computation of the shortest path graph function $spg^{s,t}$

**Input:** NCD $N = (V, C, D)$, weight assignment $W$ and $s, t \in V$
**Output:** Shortest path graph function $spg^{s,t} : V \times V \rightarrow \{0, 1\}$
**if** $dist(s, t) = \infty$ **then** $spg^{s,t}(v, v') := 0$ for all $v, v' \in V$
**else**
  **for** $v, v' \in V$ **do**
    **if** $dist(s, t) = dist(s, v) + W(v, v') + dist(v', t)$ **then** $spg^{s,t}(v, v') := 1$
    **else** $spg^{s,t}(v, v') := 0$
**return** $spg^{s,t}$

**Complexity Upper Bounds.** We present first a few useful observations. Because network connectivity can be checked independently for each source $s$ and target $t$ where $D(s, t) > 0$ by computing the maximum flow [14] between $s$ and $t$, we obtain the following lemma.

**Lemma 1.** *Given an NCD $N = (V, C, D)$ and a nonnegative integer $k$, it is polynomial-time decidable if $N$ remains connected for all sets of failed links $F \subseteq V \times V$ where $|F| \leq k$.*

Next, we present an algorithm that for an NCD $N = (V, C, D)$ with the weight assignment $W : V \times V \mapsto \mathbb{N} \cup \{\infty\}$ and a given pair of nodes $s, t \in V$ computes in polynomial time the function $spg^{s,t} : V \times V \rightarrow \{0, 1\}$ that assigns the value 1 to exactly all edges that appear on at least one shortest path (w.r.t. to the weight assignment $W$) between $s$ and $t$. The edges that get assigned the value 1 hence form the shortest path subgraph between $s$ and $t$. The algorithm uses the function $dist(v, v')$ that for every two nodes $v, v' \in V$ returns the length of the shortest path (again w.r.t. to the assignment $W$) from $v$ to $v'$ and if $v$ and $v'$ are not connected then it returns $\infty$. Such an all-pairs shortest path function can be precomputed in polynomial time using e.g. the Johnson's algorithms [27]. The function $spg^{s,t}$ is defined by Algorithm 1.

**Lemma 2.** *Let $N = (V, C, D)$ be an NCD with weight assignment $W$ and $s, t \in V$. Algorithm 1 runs in polynomial time and the value of $spg^{s,t}(v, v')$ can be returned in nondeterministic logarithmic space. Moreover, there is an edge $(v, v') \in \pi$ for some $\pi \in SPaths(s, t)$ iff $spg^{s,t}(v, v') = 1$.*

We first present results for $k = 0$ (no link failures) and start by showing that the optimistic splittable variant of the capacity problem is decidable in polynomial time by reducing it to the feasibility of a linear program. Let $N = (V, C, D)$ be an NCD with weight assignment $W$ and let $spg^{s,t}$ be precomputed for all pairs of $s$ and $t$. We construct a linear program over the variables $x^{s,t}(v, v')$ for all $s, t, v, v' \in V$ where the variable $x^{s,t}(v, v')$ represents the percentage of the total demand $D(s, t)$ between $s$ and $t$ that is routed through the link $(v, v')$. In the equations below, we let $s$ and $t$ range over all nodes that satisfy $D(s, t) > 0$.

$$1 \geq x^{s,t}(v,v') \geq 0 \quad \text{for } s,t,v,v' \in V \tag{1}$$

$$\sum_{v \in V} x^{s,t}(s,v) \cdot \mathit{spg}^{s,t}(s,v) = 1 \quad \text{for } s,t \in V \tag{2}$$

$$\sum_{v \in V} x^{s,t}(v,t) \cdot \mathit{spg}^{s,t}(v,t) = 1 \quad \text{for } s,t \in V \tag{3}$$

$$\sum_{v' \in V} x^{s,t}(v',v) \cdot \mathit{spg}^{s,t}(v',v) = \sum_{v' \in V} x^{s,t}(v,v') \cdot \mathit{spg}^{s,t}(v,v') \quad \text{for } s,t,v \in V, v \notin \{s,t\} \tag{4}$$

$$\sum_{s,t \in V} x^{s,t}(v,v') \cdot \mathit{spg}^{s,t}(v,v') \cdot D(s,t) \leq C(v,v') \quad \text{for } v,v' \in V \tag{5}$$

Equation 1 imposes that the flow portion on any link must be between 0 and 1. Equation 2 makes sure that portion of the demand $D(s,t)$ must be split along all outgoing links from $s$ that belong to the shortest path graph. Similarly Equation 3 guarantees that the flows on incoming links to $t$ in the shortest path graph deliver the total demand. Equation 4 is a flow preservation equation among all incoming and outgoing links (in the shortest path graph) connected to every node $v$. The first four equations define all possible splittings of the flow demands for all $s$ and $t$ such that $D(s,t) > 0$. Finally, Equation 5 checks that for every link in the network, the total sum of the flows for all $s$-$t$ pairs does not exceed the link capacity. The size of the constructed system is quadratic in the number of nodes and its feasibility, that can be verified in polynomial time [39], corresponds to the existence of a solution for the OS problem.

**Theorem 1.** *The OS capacity problem without any link failures is decidable in polynomial time.*

If we now restrict the variables to nonnegative intergers, we get an instance of integer linear program where feasibility checking is NP-complete [39], and corresponds to the solution for the nonsplittable optimistic problem.

**Theorem 2.** *The ON capacity problem without any link failures is decidable in nondeterministic polynomial time.*

Next, we present a theorem stating that both the splittable and nonsplittable variants of the pessimistic capacity problem are decidable in polynomial time and in fact also in nondeterministic logarithmic space (the complexity class NL).

**Theorem 3.** *The PS and PN capacity problems without any link failures are decidable in nondeterministic logarithmic space.*

*Proof.* Let $N = (V, C, D)$ be a given NCD with a weight assignment $W$. Let us consider the shortest path graph represented by $\mathit{spg}^{s,t}$ as defined by Algorithm 1. Clearly, if the set $\mathit{SPaths}(s,t)$ for some $s,t \in V$ where $D(s,t) > 0$ is empty, the answer to both the splittable and nonsplittable problem is negative. Otherwise, for each pair $s, t \in V$ where $D(s, t) > 0$, the entire demand $D(s, t)$ can be routed (both in the splittable and nonsplittable case) through any edge $(v, v')$ that satisfies $spg^{s,t}(v, v') = 1$. Hence we can check whether for every edge $(v, v') \in V \times V$ it holds

$$\sum_{\substack{s,t \in V \\ D(s,t)>0}} D(s, t) \cdot spg^{s,t}(v, v') \leq C(v, v') \ .$$

If this is the case, then the answer to both the splittable and the nonsplittable pessimistic problem is positive as there is no flow assignment that can exceed the capacity of any link. On the other hand, if for some link $(v, v')$ the sum of all demands that can be possibly routed through $(v, v')$ exceeds the link capacity, the answer to the problem (both splittable and nonsplittable) is negative. The algorithm can be implemented to run in nondeterministic logarithmic space. □

Let us now turn our attention to the four variants of the problem under the assumption that up to $k$ links can fail (where $k$ is part of the input to the decision problem). Given an NCD $N = (V, C, D)$ with a weight assignment $W$, we are asked to check, for all (exponentially many) failure scenarios $F \subseteq V \times V$ where $|F| \leq k$, whether the pruned NCD $N^F$ with the weight assignment $W^F$ (as defined in Definition 3) satisfies that the network $N^F$ is connected and every flow assignment is feasible (in case of the pessimistic case) or there exists a feasible flow assignment (in case of the optimistic case). As these problems are decidable in polynomial time for PN, PS and OS, we can conclude that the variants of the problems with failures belong to the complexity class co-NP: for the negation of the problems we can guess the failure scenario $F$ for which the problem does not have a solution—this can be verified in polynomial time by Theorems 1 and 3.

**Theorem 4.** *The PN, PS and OS problems with link failures are in co-NP.*

Finally, the same arguments can be used also for the optimistic nonsplittable problem with failures. However, as deciding the ON problem without failures is solvable only in nondeterministic polynomial time, the extra quantification of all failure scenarios means that the problem belongs to the class $\Pi_2^P$ on the second level of the polynomial hierarchy [33]. This complexity class is believed to be computationally more difficult than the problems on the first level of the hierarchy (where the NP and co-NP problems belong to).

**Theorem 5.** *The ON problem with link failures is in the complexity class $\Pi_2^P$.*

**Complexity Lower Bounds.** We now prove the complexity lower bounds.

**Theorem 6.** *The OS capacity problem without any link failures is P-hard under NC-reducibility.*

*Proof sketch.* By NC-reduction from the P-complete maximum flow problem for directed acyclic graphs [35]: given a directed acyclic graph $G$ with nonnegative edge capacities, two nodes $s$ and $t$ and a number $m$, is there a flow between $s$ and $t$ that preserves the capacity of all edges and has the volume of at least $m$? This problem can be rephrased as our OS problem by setting the demand $D(s,t) = m$ and defining a weight assignment so that every relevant edge in $G$ is on some shortest path from $s$ to $t$. This can be achieved by topologically sorting the nodes (in $NC^2$ [11,12]) and assigning the weights accordingly. □

**Theorem 7.** *The PS/PN problems without any link failures are NL-hard.*

*Proof sketch.* Follows from NL-hardness of reachability in digraphs [33]. □

Next, we show that the ON problem is NP-hard, even with no failures.

**Theorem 8.** *The ON capacity problem without any link failures is NP-hard, even for the case where all weights are equal to 1.*

*Proof.* By a polynomial-time reduction from the NP-complete problem CNF-SAT [33]. Let $\varphi = c_1 \wedge c_2 \wedge \ldots \wedge c_n$ be a CNF-SAT instance where every clause $c_i$, $1 \leq i \leq n$, is a disjunction of literals. A literal is either a variable $x_1, \ldots, x_k$ or its negation $\overline{x}_1, \ldots, \overline{x}_k$. If a literal $\ell_j \in \{x_j, \overline{x}_j\}$ appears in the disjunction for the clause $c_i$, we write $\ell_j \in c_i$. A formula $\varphi$ is *satisfiable* if there is an assignment of the variables $x_1, \ldots, x_k$ to true or false, so that the formula $\varphi$ is satisfied (evaluates to true under this assignment). For a given formula $\varphi$ we now construct an NCD $N = (V, C, D)$ where

- $V = \{s_0, s_1, \ldots, s_k\} \cup \{x_1, \ldots, x_k\} \cup \{\overline{x}_1, \ldots, \overline{x}_k\} \cup \{c_i^s, c_i^e \mid 1 \leq i \leq n\}$,
- $C(s_{i-1}, x_i) = C(s_{i-1}, \overline{x}_i) = C(x_i, s_i) = C(\overline{x}_i, s_i) = n$ for all $i$, $1 \leq i \leq k$,
- $C(c_i^s, \ell_j) = C(s_j, c_i^e) = 1$ for all $i$, $1 \leq i \leq n$ and every literal $\ell_j \in \{x_j, \overline{x}_j\}$ such that $\ell_j \in c_i$,
- $D(s_0, s_k) = n$, and $D(c_i^s, c_i^e) = 1$ for all $i$, $1 \leq i \leq n$.

The capacities of edges and flow demands that are not mentioned above are all set to 0 and the weights of all edges are equal to 1. In Figure 4a we give an example of the reduction for a given satisfiable formula. As we consider the nonsplittable problem, the flow demand from $s_0$ to $s_k$ means that the whole demand of $n$ units must go through either the link $(x_i, s_i)$ or $(\overline{x}_i, s_i)$, for every $i$. This corresponds to choosing an assignment of the variables to true or false. For every clause $c_i$ we now have a unit flow from $c_i^s$ to $c_i^e$ that goes through the link $(\ell_j, s_j)$ for every literal $\ell_j$ appearing in the clause $c_i$. This is only possible if this link is not already occupied by the flow demand from $s_0$ to $s_k$; otherwise we exceed the capacity of the link. For each clause $c_i$ we need to find at least one literal $\ell_j$ so that the flow can go through the edge $(\ell_j, s_j)$. As the capacity of the edge $(\ell_j, s_j)$ is $n$, it is possible to use this edge for all $n$ clauses if necessary. We can observe that the capacity network can be constructed in polynomial time and we shall argue for the correctness of the reduction.

We can now observe that if $\varphi$ is satisfiable, we can define a feasible flow assignment $f$ by routing the flow demand of $n$ between $s_0$ and $s_k$ so that it does

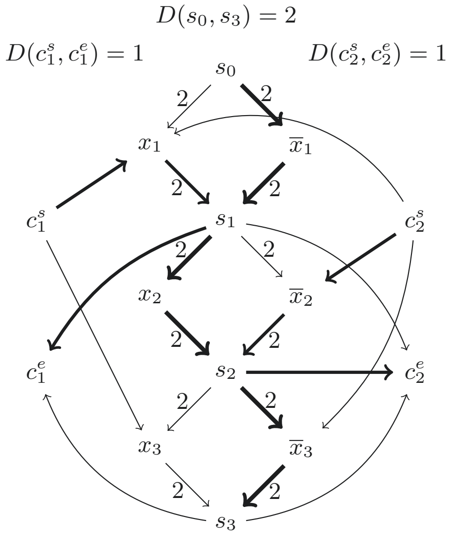

(a) NCD for the formula $(x_1 \vee x_3) \wedge (x_1 \vee \overline{x}_2 \vee \overline{x}_3)$. The capacity of unlabelled links is 1, otherwise 2; link weights are 1. Thick lines show a feasible nonsplittable flow assignment.

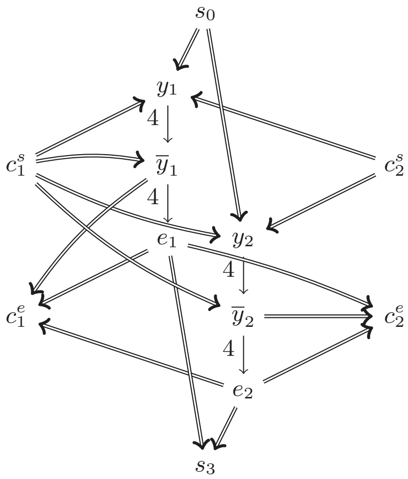

(b) Additional construction for the formula $\forall y_1, y_2.\ \exists x_1, x_2, x_3.\ (x_1 \vee x_3 \vee y_1 \vee \overline{y}_1 \vee \overline{y}_2) \wedge (x_1 \vee \overline{x}_2 \vee \overline{x}_3 \vee y_2)$. Capacity of all links is 4 and weight of links is 1. Double arrows are 2-unbreakable links.

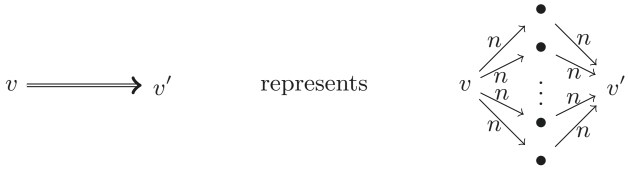

(c) Definition of $m$-unbreakable link of capacity $n$ with $m + 1$ intermediate nodes

Fig. 4: Reduction to ON capacity problem without/with failures not use the links corresponding to the satisfying assignment for $\varphi$ and then every clause in $\varphi$ can be routed through the links corresponding to one of the satisfied literals. For the other direction where $\varphi$ is not satisfieable, we notice that any routing of the flow demand between $s_0$ and $s_k$ (corresponding to some truth assignment of $\varphi$) leaves at least one clause unsatisfied and it is then impossible to route the flow for such a clause without violating the capacity constraints. $\square$

We now extend the reduction from Theorem 8 to the OS case with link failures and prove its hardness for the second level of the polynomial hierarchy.

**Theorem 9.** *The ON problem with link failures is $\Pi_2^P$-hard.*

*Proof.* By reduction from the validity of the quantified Boolean formula of the form $\forall y_1, y_2, \ldots, y_m.\ \exists x_1, x_2, \ldots, x_k.\ \varphi$ where $\varphi = c_1 \wedge c_2 \wedge \ldots \wedge c_n$ is a Boolean formula in CNF over the variables $y_1, \ldots, y_m, x_1, \ldots, x_k$. The validity problem of such quantified formula is $\Pi_2^P$-hard (see e.g. [33]). For a given quantified formula, we shall construct an instance of the ON problem such that the formula is valid if and only if the ON problem with up to $m$ link failures (where $m$ is the number of $y$-variables) has a positive answer. The reduction uses the construction from Theorem 8 where we described a reduction from the validity of the formula $\exists x_1, x_2, \ldots, x_k.\ \varphi$. The construction is further enhanced by introducing new nodes $y_j, \overline{y}_j, e_j$ and new edges of capacity $2n$ (where $n$ is the number of clauses) such that $C(y_j, \overline{y}_j) = C(\overline{y}_j, e_j) = 2n$, for all $i$, $1 \leq j \leq m$.

Now for every clause $c_i$ we add the so-called $m$-unbreakable edge of capacity $n$ from $c_i^s$ to $y_j$ and from $e_j$ to $c_i^e$ for all $1 \leq i \leq n$ and $1 \leq j \leq m$. Moreover, whenever the literal $y_j$ appears in the clause $c_i$, we also add an $m$-unbreakable edge from $\overline{y}_j$ to $c_i^e$ and whenever the literal $\overline{y}_j$ appears in the clause $c_i$, we add $m$-unbreakable edge from $c_i^s$ to $\overline{y}_j$. The construction of $m$-unbreakable edges (denoted by double arrows) is given in Figure 4c where the capacity of each link is set to $n$. Finally, for each $j$, $1 \leq j \leq m$, we add the unbreakable edges from $s_1$ to $y_j$ and from $e_j$ to $s_k$. The flow demands in the newly constructed network are identical to those from the proof of Theorem 8 and the weights of all newly added edges are set to 1 and we set the weight of the two links $s_0$ to $x_1$ and $s_0$ to $\overline{x}_1$ to 6. The reduction can be clearly done in polynomial time. Figure 4b demonstrates an extension of the construction from Figure 4a with additional nodes and links that complete the reduction. Observe, that even in case of $m$ link failures, the unbreakable links that consist of $m+1$ edge disjoint paths are still capable of carrying all the necessary flow traffic.

We shall now argue that if the formula $\forall y_1, y_2, \ldots, y_m.\ \exists x_1, x_2, \ldots, x_k.\ \varphi$ is valid then the constructed instance of the ON problem with up to $m$ link failures has a solution. We notice that any subset of up to $m$ failed links either breaks exactly one of the newly added edges $(y_j, \overline{y}_j)$ and $(\overline{y}_j, e_j)$ for all $j$, $1 \leq j \leq m$, in which case this determines a valid truth assignment for the $y$-variables and as in the previous proof, the flow from $s_0$ to $s_k$ can now be routed so that for each clause there is at least one satisfied literal. Otherwise, there is a variable $y_j$ such that both of the edges $(y_j, \overline{y}_j)$ and $(\overline{y}_j, e_j)$ are present and all flow demands can now be routed through these two edges (that have sufficient capacity for this) by using the $m$-unbreakable edges. The opposite direction where the formula is not valid means that there is a truth assignment to the $y$-variables so that irrelevant of the assignment for $x$-variables there is at least one clause that is not satisfied. We simply fail the edges that correspond to such a $y$-variables assigment and the same arguments as in the previous proof imply that there is not any feasible flow assignment for this failure scenario. □

**Theorem 10.** *The PN, PS and OS problems with link failures are co-NP-hard.*

*Proof sketch.* By reduction from the NP-complete *shortest path most vital edges* problem (SP-MVE) [3,36]. The input to SP-MVE is a directed graph $G = (V, E)$ with positive edge weights, two nodes $s, t \in V$ and two positive numbers $k$ and

**Algorithm 2** Brute-force search

**Input:** NCD $N = (V, C, D)$ with weigth assignment $W$, a number $k \geq 0$ and type of the capacity problem $\tau \in \{\mathrm{PS}, \mathrm{PN}, \mathrm{ON}, \mathrm{OS}\}$
**Output:** *true* if the answer to the $\tau$-problem is positive, else *false*
**for all** $F \subseteq V \times V$ s.t. $|F| \leq k$ and $C(v, v') > 0$ for all $(v, v') \in F$ **do**
  construct network $N^F$ and weight assignment $W^F$ by Definition 3
  **switch** $\tau$ **do**
    **case** OS: use Theorem 1 on $N^F$ and $W^F$ (without failed links)
    **case** ON: use Theorem 2 on $N^F$ and $W^F$ (without failed links)
    **case** PS/PN: use Theorem 3 on $N^F$ and $W^F$ (without failed links)
  **if** the answer to the $\tau$-problem on $N^F$ and $W^F$ is negative **then return** *false*
**endfor**
**return** *true*

$H$. The question is whether there exist at most $k$ edges in $E$ such that their removal creates a graph with the length of the shortest path between $s$ and $t$ being at least $H$. We reduce the SP-MVE to the negation of the PN/PS in order to demonstrate co-NP-hardness.

We modify the $G$ by inserting a new edge between $s$ and $t$ of weight $H$ and capacity 1, while setting the capacity 2 for all other edges in $G$. If the SP-MVE problem has a solution $F \subseteq E$ where $|F| \leq k$, then the added edge $(s, t)$ becomes one of the shortest paths between $s$ and $t$ under the failure scenario $F$ and a flow demand of size 2 between $s$ and $t$ can be routed through this edge, violating the capacity constraints. If the SP-MVE problem does not have a solution, then after the removal of at most $k$ links, the length of the shortest path between $s$ and $t$ remains strictly less than $H$ and any flow assignment along the shortest paths is feasible. We hence conclude that PN/PS problems are co-NP-hard. A small modification of the construction is needed for hardness of the OS problem. □

## 4 A Fast Strategic Search Algorithm

In order to solve the PS, PN, ON and OS problems, we can enumerate all failure scenarios for up to $k$ failed links (omitting the links with zero capacity), construct the pruned network for each such failure scenario and then apply our algorithms in Theorems 1, 2 and 3. This brute-force search approach is formalized in Algorithm 2 and its worst-case running time is exponential.

Our complexity results indicate that the exponential behavior of any algorithm solving a co-NP-hard (or even $\Pi_2^P$-hard) problem is unavoidable (unless P=NP). However, in practice many concrete instances can be solved fast if more refined search algorithms are used. To demonstrate this, we present a novel strategic search algorithm for verifying the feasibility of shortest path routing under failures. At the heart of our algorithm lies the idea to reduce the number of explored failure scenarios by skipping the "uninteresting" ones. Let us fix an NCD $N = (V, C, D)$ with the weight assignment $W$. We define a relation $\prec$ on failure scenarios such that $F \prec F'$ iff for all flow demands we preserve in $F'$ at least one of the shortest paths that are present under the failure scenario $F$.

**Definition 9.** *Let $F, F' \in V \times V$. We say that $F$ preceeds $F'$, written $F \prec F'$, if $\mathit{SPaths}^F(s,t) \supseteq \mathit{SPaths}^{F'}(s,t)$ and $\mathit{SPaths}^F(s,t) \cap \mathit{SPaths}^{F'}(s,t) \neq \emptyset$ for all $s, t \in V$ where $D(s,t) > 0$.*

We first show that if $F \prec F'$ and the failure scenario $F$ has a feasible routing solution for the pessimistic problem, then $F'$ also has a solution. Thus instead of exploring all possible failure scenarios like in the brute-force algorithm, it is sufficient to explore only failure scenarios that are minimal w.r.t. $\prec$ relation.

**Lemma 3.** *Let $F, F' \in V \times V$ where $F \prec F'$. A positive answer to the PS/PN problem for the network $N^F$ with weight assignment $W^F$ implies a positive answer to the PS/PN problem for the network $N^{F'}$ with weight assignment $W^{F'}$.*

For the optimistic scenario, the implication is valid in the opposite direction: it is sufficient to explore only the maximum failure scenarios w.r.t. $\prec$.

**Lemma 4.** *Let $F, F' \in V \times V$ where $F \prec F'$. A positive answer to the OS/ON problem for the network $N^{F'}$ with weight assignment $W^{F'}$ implies a positive answer to the OS/ON problem for the network $N^F$ with weight assignment $W^F$.*

Hence for the pessimistic scenario, the idea of strategic search is to ignore failure scenarios that remove only some of the shortest paths but preserve at least one of such shortest paths. For the optimistic scenario, we on the other hand explore only the maximal failure scenarios where removing one additional link causes the removal of all shortest paths for at least one source and destination.

In our algorithm, we use the notation $\mathit{spg}_F^{s,t}$ for the shortest path graph as defined in Algorithm 1 for the input graph $N^F$ with weight assignment $W^F$. The function $\mathit{min\_cuts}(\mathit{spg}_F^{s,t}, s, t)$ returns the set of all minimum cuts separating the nodes $s$ and $t$ (sets of edges that disconnect the source node $s$ from the target node $t$ in the shortest-path graph $\mathit{spg}_F^{s,t}$). This function can be computed e.g. using the Provan and Shier algorithm [34], assuming that each edge has a unit weight and hence minimizing the number of edges in the minimum cut. There can be several incomparable minimum cuts (with the same number of edges) and by $\mathit{mincut\_size}(\mathit{spg}_F^{s,t}, s, t)$ we denote the number of edges in each the minimum cuts from the set $\mathit{min\_cuts}(\mathit{spg}_F^{s,t}, s, t)$.

Algorithm 3 now presents our fast search strategy, called *strategic search*. The input to the algorithm is the same as for the brute-force search. The algorithm initializes the *pending* set of failure scenarios to be explored to the empty failure scenario and it remembers the set of *passed* failure scenarios that were already verified. In the main while loop, a failure scenario $F$ is removed from the *pending* set and depending on the type $\tau$ of the problem, we either directly verify the scenario $F$ in the case of the pessimistic problems, or we call the function *MaxFailureCheck*$(F)$ that instead verifies all maximal failure scenarios $F'$ such that $F \prec F'$. The correctness of Algorithm 3 is formally stated as follows.

**Theorem 11.** *Algorithm 3 terminates and returns true iff the answer to the $\tau$-problem is positive.*

**Algorithm 3** Strategic search

**Input:** NCD $N = (V, C, D)$ with weigth assignment $W$, a number $k \geq 0$ and type of capacity problem $\tau \in \{\text{PS}, \text{PN}, \text{ON}, \text{OS}\}$
**Output:** *true* if the answer to the $\tau$-problem is positive, else *false*
$pending := \{\emptyset\}$ * initialize the pending set with the empty failure scenario *\
$passed := \emptyset$ * already processed failure scenarios *\
**while** $pending \neq \emptyset$ **do**
    **let** $F \in pending$; $pending := pending \setminus \{F\}$
    **switch** $\tau$ **do**
        **case** $\tau \in \{\text{PS}, \text{PN}\}$: Build $N^F$ and $W^F$ by Definition 3, use Theorem 3
            **if** the answer to the $\tau$-problem was negative **then return** *false*
        **case** $\tau \in \{\text{OS}, \text{ON}\}$: **call** $\mathit{MaxFailureCheck}(F)$
    $passed := passed \cup \{F\}$
    **for** $s, t \in V$ such that $D(s, t) > 0$ **do**
        **if** $|F| + \mathit{mincut\_size}(spg_F^{s,t}, s, t) \leq k$ **then**
            $succ := \{F \cup C \mid C \in \mathit{min\_cuts}(spg_F^{s,t}, s, t),\ F \cup C \notin (pending \cup passed)\}$
            $pending := pending \cup succ$
**endwhile**
**return** *true*

**procedure** $\mathit{MaxFailureCheck}(F)$ * to be run only for the optimistic cases *\
**for** $s, t \in V$ such that $D(s, t) > 0$ **do**
    **for** $C \in \mathit{min\_cuts}(spg_F^{s,t}, s, t)$ **do**
        **for all** $C' \subset C$ such that $|F \cup C'| = \min(k, |F \cup C| - 1)$ **do**
            **if** $F \cup C' \notin passed$ **then**
                construct $N^{F \cup C'}$ and $W^{F \cup C'}$ by Definition 3
                **switch** $\tau$ **do**
                    **case** $\tau = \text{OS}$: use Theorem 1 and **if** negative **then return** *false*
                    **case** $\tau = \text{ON}$: use Theorem 2 and **if** negative **then return** *false*
                $passed := passed \cup \{F \cup C'\}$
        **endfor**
    **endfor**
**endfor**

## 5 Experiments

To evaluate the practical performance of our strategic search algorithms, we conducted experiments on various wide-area and datacenter network topologies. The reproducibility package with our Python implementation can be found at [37].

We study the algorithms' performance on a range of network topologies, and consider both sparse and irregular wide-area networks (using the Internet Topology Zoo [28] data set) as well as dense and regular datacenter topologies (namely fat-tree [9], BCube [23], and Xpander [40]). To model demands, for each topology, we consider certain nodes to serve as core nodes which have significant pairwise demands. Overall, we created 24,388 problem instances for our experimental benchmark, out of which we were able to solve 23,934 instances

| Topology | Problem | B.iter | B.time | S.iter | S.time | Speedup |
|---|---|---|---|---|---|---|
| BCube | ON | 105 | 79.5 | 1 | 1.7 | 47.1 |
| BCube | OS | 2081 | 348.2 | 768 | 125.1 | 2.8 |
| BCube | PS/PN | 5051 | 170.0 | 1 | 0.1 | 4684.0 |
| Fat-tree | ON | 105 | 59.4 | 1 | 1.2 | 47.6 |
| Fat-tree | OS | 41 | 2.0 | 1 | 0.2 | 8.5 |
| Fat-tree | PS/PN | 43745 | 562.6 | 1 | 0.1 | 66976.3 |
| Xpander | ON | 254 | 407.3 | 1 | 3.0 | 137.7 |
| Xpander | OS | 170 | 124.1 | 1 | 1.6 | 78.0 |
| Xpander | PS/PN | - | >7200.0 | 1 | 5.4 | >1340.6 |
| Topology Zoo | ON | 127 | 59.6 | 8 | 4.6 | 12.9 |
| Topology Zoo | OS | 596 | 35.3 | 46 | 2.6 | 13.4 |
| Topology Zoo | PS/PN | 86 | 4.3 | 2 | 0.1 | 82.7 |

Fig. 5: Median results, time in seconds (**B**: brute-force search, **S**: strategic search)

within a 2-hour timeout. In our evaluation, we filter out the trivial instances where the runtime is less than 0.1 second for both the brute-force and strategic search (as some of the instances e.g. contain a disconnected flow demand already without any failed links). The benchmark contains a mixture of both positive and negative instances for each problem for increasing number $k$ of failed links.

Table 5 shows the median times for each series of experiments for the different scenarios. All experiments for each topology and given problem instance are sorted by the speedup ratio, i.e. B.time divided by S.time; we display the result for the experiment in the middle of each table. Clearly, our strategic search algorithm always outperforms the brute-force one by a significant factor in all the scenarios. We also report on the number of iterations (B.iter and S.iter) of the two algorithms, showing the number of failure scenarios to be explored.

Let us first discuss the pessimistic scenarios in more detail. Figure 6 shows a cactus plot [6] for the wide-area network setting (on the left) and for the datacenter setting (on the right). We note that y-axis in the figure is logarithmic. For example, to solve the 1500th fastest instances in the wide-area network (left), the brute-force algorithm uses more than 100 seconds, while the strategic algorithm solves the problem in less than a second; this corresponds to a speedup of more than two orders of magnitude. For more difficult instances, the difference in runtime continues to grow exponentially, and becomes several orders of magnitude. For datacenter networks (right), the difference is even larger. The latter can be explained by the fact that datacenters provide a higher path diversity and multiple shortest paths between source and target nodes and hence more opportunities for a clever skipping of "uninteresting instances". As the pessimistic problems we aim to solve are co-NP-hard, there are necessarily some hard instances also for our strategic search; this is demonstrated by the S-shaped curve showing a significantly increased runtime for the most difficult instances.

We next discuss the optimistic scenarios, including the experiments both for splittable and nonsplittable cases. Figure 7 shows a cactus plot for the wide-area

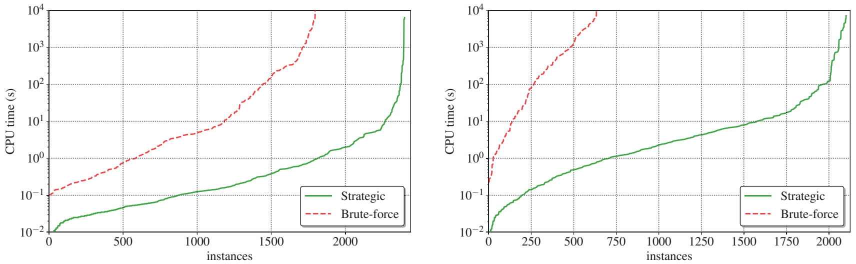

Fig. 6: Pessimistic scenario. Left: wide-area networks, right: datacenter networks

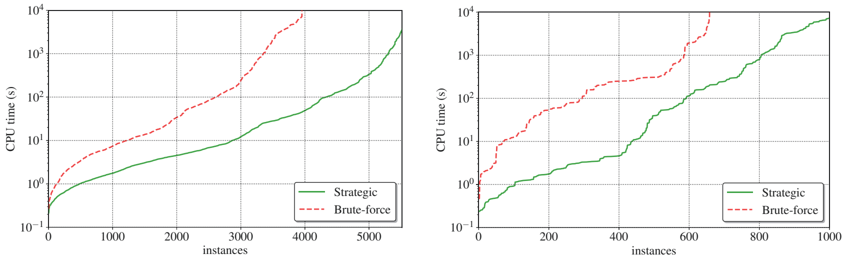

Fig. 7: Optimistic scenario. Left: wide-area networks, right: datacenter networks network setting (on the left) and for the datacenter setting (on the right). Again, our strategic algorithm significantly outperforms the baseline in both scenarios. Interestingly, in the optimistic scenario, the relative performance benefit is larger for wide-area networks as the optimistic strategic search explores all the maximum failure scenarios and there are significantly more of such scenarios in the highly connected datacenter topologies. Hence, while for datacenters (right) the strategic search maintains about one order of magnitude better performance, the performance for the wide-area networks improves exponentially.

## 6 Conclusion

We presented a comprehensive study of the algorithmic complexity of verifying feasible routes under failures without violating capacity constraints, covering both optimistic and pessimistic, as well as splittable and nonsplittable scenarios. We further presented algorithms, based on strategic failure scenario enumerations, which we proved efficient in realistic scenarios. While our paper charts the complete landscape, there remain several interesting avenues for future research like further scalability improvements and a parallelization of the algorithm.

*Acknowledgements.* Research supported by the Vienna Science and Technology Fund (WWTF) project ICT19-045 and by the DFF project QASNET.
